# Acoustic Performance of Resilient Materials Using Acrylic Polymer Emulsion Resin

**DOI:** 10.3390/ma9070592

**Published:** 2016-07-19

**Authors:** Haseog Kim, Sangki Park, Seahyun Lee

**Affiliations:** Building and Urban Research Institute, Korea Institute of Civil Engineering and Building Technology, 283, Goyang-daero, Ilsanseo-gu, Goyang-si, Gyeonngi-do 10223, Korea; bravo3po@kict.re.kr (H.K.); shlee@kict.re.kr (S.L.)

**Keywords:** heavyweight impact sound, resilient material, acrylic polymer emulsion resin, floor impact sound reduction, floor system

## Abstract

There have been frequent cases of civil complaints and disputes in relation to floor impact noises over the years. To solve these issues, a substantial amount of sound resilient material is installed between the concrete slab and the foamed concrete during construction. A new place-type resilient material is made from cement, silica powder, sodium sulfate, expanded-polystyrene, anhydrite, fly ash, and acrylic polymer emulsion resin. Its physical characteristics such as density, compressive strength, dynamic stiffness, and remanent strain are analyzed to assess the acoustic performance of the material. The experimental results showed the density and the dynamic stiffness of the proposed resilient material is increased with proportional to the use of cement and silica powder due to the high contents of the raw materials. The remanent strain, related to the serviceability of a structure, is found to be inversely proportional to the density and strength. The amount of reduction in the heavyweight impact noise is significant in a material with high density, high strength, and low remanent strain. Finally, specimen no. R4, having the reduction level of 3 dB for impact ball and 1 dB for bang machine in the single number quantity level, respectively, is the best product to obtain overall acoustic performance.

## 1. Introduction

Recently, the average annual rate of increase in multi-unit dwellings in South Korea was recorded at 5.6% and despite the decline in the percentage increase resulting from low national growth, their housing market share has steadily been on the rise. Those living in multi-unit dwellings, which are the most common type of housing in South Korea, are likely to be exposed to noise from dwelling as the walls, floors, and ceilings are shared by the vertically and horizontally adjacent housing units. With a growing demand for improved quality of life, there have been frequent cases of civil complaints and disputes in relation to floor impact noises. To be more specific, the number of disputes filed due to floor impact noise more than doubled from 7021 cases in 2012 to 15,485 cases in 2013 [1,2,3]. In an effort to resolve this issue, the government of South Korea has revised the Criteria for the interlayer floor impact sound regulation in multi-family residential housing [4]. According to the revised standards, the standard floor system is to be excluded, and a floor impact noise performance rating certification institution is to check the performance of the insulation system and introduce a certified floor system to judge the conformity of the system in relation to the noise insulation performance. It should be noted that the noise insulation performance is defined as 43 dB (A) for direct impact noise in day time and 38 dB (A) for night time, and 45 dB (A) for airborne noise in day time and 40 dB (A) for night time at single number quantity (SNQ) with A-weighting. It also prescribes that a slab thickness of at least 160 mm has to be maintained for the frame structure, while in the case of other structures (walls, flat plates, mixed structures, etc.), additional experiments are to be conducted for a performance rating of the insulation against light- and heavyweight floor impact noise [5].

Researches related to obtaining the best acoustic performance in buildings can be divided into two categories: one is to develop a new material and a floor system to implement higher acoustic insulation in buildings and the other is to exploit testing methods to characterize their acoustic performance. Branco and Godinho [6] considered various types of lightweight mortars, which are cement mortars containing expanded polystyrene, expanded cork, and expanded clay granulates, to investigate acoustic performance. The acoustic characteristics of lightweight mortars were evaluated through laboratory tests using small-size acoustic chamber. From the results, a sensible impact sound reduction without any floor covering was obtained, especially for higher frequencies. Martins et al. [7] determined the acoustic behavior of different constructive solutions based on two types of floor, i.e., timber and timber–concrete floor systems. The development of a new floor system is considered an alternative way to improve acoustic performance of a building. 

Brancher et al. [8] evaluated the acoustical performance of the proposed polymer waste based on the mortar. Thickness and replacement percentage of the natural aggregate by EVA (Ethylene Vinyl Acetate) were considered. Results of the impact noise test showed that the performance of 50% EVA replacement product reached an impact sound insulation of 23 dB compared to uncovered slab. They concluded that polymer waste addition decreased the mortar compressive strength, and EVA displayed characteristics of an influential material to intensify other features of the composite.

D’Alessandro et al. [9] studied a sustainable lightweight concrete containing polymers derived from the recycling of sheets of electric wires. They measured structural properties of the sustainable lightweight concretes, i.e., dynamic stiffness, impact sound pressure reduction, and thermal conductivity. Finally, they concluded that the developed concrete can be successfully used for thermal and acoustic insulating lightweight screeds to be applied above the concrete structural slabs in floors. In addition, Antonio et al. [10] developed a lightweight cement-based screeds containing cork granule waste. Acoustic performance of the proposed screeds was measured with/without a resilient layer between the heavyweight standard floor and a floating concrete layer. Three different screeds and three thicknesses were considered. It concluded that the impact sound reduction of resilient materials is related to their dynamic stiffness and results of dynamic stiffness are generally found to be related to the values of impact sound reduction when the cement/cork screed is used as floor covering. Kim et al. [2] stated that the installation of lightweight and low stiffness materials between the concrete slab and the finishing covering can reduce a floor impact noise in buildings. Schiavi et al. [11] investigated the acoustical performance and mechanical properties, such as dynamic stiffness, compressibility, and compressive creep, of different types of resilient materials in order to reduce transmitted impact noise. They also studied airflow resistivity as a physical parameter characterizing porous and fibrous sound absorbent materials. The evaluation of the dynamic stiffness of the porous and fibrous sound absorbent materials used as underlay in floating floor is needed. Experiments showed that airflow resistivity depends on the density increase, induced by static load, of the porous or fibrous materials [12].

Maderuelo-Sanz et al. [13] introduced a new recycled material made from granulated rubber for reducing impact noise and experimentally investigated the acoustical performance. Then the performance was compared to those of commercial products. Caniato et al. [14] conducted twenty different layers to evaluate the time-depending performance, dynamic stiffness, compressibility, and compressive creep. Experimental results indicated that the presence of coating, different density, and contact shape has been proven to influence the acoustic performance. Baron et al. [15] investigated that the performance of the floating floor depends on the mechanical properties of the insulation layer, e.g., the dynamic stiffness and mass per unit area. During the experiments, different excitation methods such as the use of a sine sweep, white noise or impact hammer were considered as an excitation signal. From the results, it is advisable to use a sine sweep excitation, as it is better than the other methods. Stewart et al. [16] proposed a simplified approach allowing the dynamic stiffness of resilient layers. The proposed method gives the stiffness across a frequency range. Reasonable agreements with measurements are obtained. Cho [17] introduced a method for measuring the creep-induced change of the dynamic stiffness. The proposed method can assess the creep-induced change of the dynamic stiffness and could be helpful to design a vibration isolation system. Monte et al. [18] studied cork having a low density can be considered as a hydrophobic and viscoelastic materials with good thermal and acoustic insulation properties. A new polymer of inorganic oxides and cork composite with improved thermal and acoustic properties is reported. 

Still, many researches have been conducted to solve the floor impact noise by utilizing experimental approaches, installing resilient materials, or considering a systematic analysis of many elements such as impact source, structural properties, etc. A major challenge in this research is that floor impact noise is influenced by a number of factors including slab thickness, spatial arrangement and indoor living space area [19,20]. Neves e Sousa and Gibbs proposed an analytical model in order to investigate the effects on impact sound transmission at low frequencies considering the location of the impact, type of floor, structural boundary conditions, floor and room dimensions, position of the receiver and room absorption [21]. You and Jeon performed both finite element simulations and field measurements in order to investigate the effects of resilient isolators and viscoelastic resilient materials on suppressing floor impact noise. Results showed that the impact vibration acceleration level and floor impact sounds at low frequencies is significantly decreased due to the installation of resilient materials, whereas the impact sound pressure levels at low frequencies were increased as a result of the use of resilient isolators [3]. Park et al. [22] studied low-frequency impact sound transmission using a finite element vibration model and an experimental sound field using a rubber ball as an impact source in order to determine the influencing factors. The results indicated that the natural frequency of the floating structures influences low frequency impact sound insulation in that impact energy is attenuated above the natural frequency. In addition, the wave fields of the floating and base plates are coupled below the natural frequency, and dominate the impact sound fields. Recently, Park and Kim [23] proposed an analytical impact force model for both bang machine and impact ball based on the actual measurements. The analytical force model was verified through a comparative review with Korean standards.

Accordingly, in order to resolve these issues, multiple measures such as use of hollow slabs have been proposed. There are economic and technical limitations in suppressing floor impact noise solely by increasing the thickness of the floor plate [24,25,26]. Under these present circumstances, a resilient material is placed in between a concrete slab and lightweight foamed concrete to eliminate a substantial amount of floor impact noise. Detailed information related to the floor system adopted in this study will be discussed later. However, the currently used resilient materials are made with organic materials with low densities and dynamic stiffness such as EPS (Expanded Polystyrene) and EVA, and when these materials are placed in between the mineral-based concrete slab and foamed concrete, floating floors start to deform due to the use of heterogeneous materials. Moreover, the slab and resilient material do not completely adhere together when such resilient materials are installed on the top of the concrete slab. This can cause problems of resonance and amplification of certain frequencies in the event of impact on the upper part. There have been reports of cracking and settlement in the finish mortar caused by upper load, due to the varying densities of the resilient material and varying shapes of the floor plate (corrugated, embossed, flat, etc.). It has also been reported that the use of a ceramic resilient material such as glass wool or mineral wool effectively reduces floor impact noise, as it enhances strength and creates an integral floor structure, thereby inducing composite behavior [2]. However, these materials, except mineral wool, are costly and exhibit high thermal conductivity, which limits their use. In order to mitigate the floor noise issues related to the use of resilient materials that are different from the floor structure, there is a need to develop a ceramic, mineral-based resilient material that can prevent resonance, enhance high stiffness, and have low thermal conductivity and high durability by mixing ceramic resins and mineral materials. Therefore, there is a great need to develop new resilient materials that can reduce floor impact noise after being installed to the floor system.

In this study, ceramic resins and mineral binders were selected and mixed together to fabricate a resilient material. Then, its physical characteristics such as density, dynamic stiffness, and remanent strain as well as the heavyweight sound noise properties were investigated. It should be commented that there are two types of dynamic stiffness: one is the dynamic stiffness and its unit is N/m and the other is the dynamic stiffness per area and its unit is MN/m/m^2^, i.e., MN/m^3^ or MPa/m. In this study, the dynamic stiffness per area is used. Additionally, the natural frequencies of the proposed materials were evaluated using two-plate system. Two octave bands, i.e., 1/3 Octave bands and 1/1 Octave bands, were used to evaluate the acoustic performance of the material. Finally, its effectiveness of the floor impact noise reduction has been assessed after its application to a floor system.

## 2. Experimental Test for the Material Properties

### 2.1. Test Specimens

A new place-type resilient material has been made from acrylic polymer emulsion resin, mineral binders, cement, fly ash, silica power, EPS beads, etc. The experimental test has been performed to evaluate the physical characteristics of the proposed resilient material, such as density, compressive strength, dynamic stiffness, and remanent strain. It should be noted that a common resilient material is manufactured in predetermined size and installed on the floor system in the construction site. However, the “place-type” resilient material is blending in the construction field then pouring on the floor system. Three replacement ratios for each cement and silica powder are considered in this study, respectively.

Acrylic polymer emulsion resin has low density and low dynamic stiffness and is capable of resilient and elastic behavior. Therefore, a commercial waterborne acrylic polymer emulsion resin was selected and mixed with several mineral materials. The acrylic resin used in this experiment was waterborne acrylic polymer emulsion resin that was milky white in color. Then, Type I Ordinary Portland Cement (OPC) was added to enhance stiffness and allow bearing of load, and sodium sulfate was mixed in to allow early development of strength. During the mix process, shrinkage cracks occur due to the hardening of cement. Thus, Type II anhydrite, which is grayish white in color and 550 m^2^/kg in specific surface area, was used as an expansion agent to prevent them. Then, Type II fly ash, which has an ignition loss of 4.87%, density of 434.78 kg/m^3^, and fineness of 452 m^2^/kg, was added to suppress the initial heat of hydration, reduce contraction and enhance long-term strength. Sodium sulfate (Na_2_SO_4_) used to promote early strength development was comprised of 43.0% Na_2_O and 55.5% SO_3_, while the silica powder had a purity of 99.8% (SiO_2_) and fineness of 2100 m^2^/kg. In addition, silica powder was used as a filler to increase density and to inhibit shrinkage cracks resulting from the hardening of acrylic resin. A specified amount of EPS beads were added to reduce the thermal conductivity of the resilient materials so as to satisfy the insulation performance. A replacement ratio of each component for the proposed resilient materials is shown in Table 1.

One can see that the summation of all six components is not equal to 100%. This is because the replacement ratio in Table 1 is the weight for each component when the weight for the acrylic polymer emulsion resin, which is considered to be the base component, is assumed to be 100%. Therefore, the actual weight of each component in these specimens has to be computed based on the consideration of the weight for the acrylic polymer emulsion resin and the replacement ratio in Table 1. For instance, the summation of all six components for M5 specimens is only 85%, i.e., the weight for cement is 15%, 30% for silica powder, 1% for both sodium sulfate and EPS, 8% for anhydrite, and 30% for fly ash. Then, the weight for acrylic polymer emulsion resin, assumed to be 100%, must be added. Therefore, the summation of all seven components is 185%, i.e., 85% + 100%. Then, the weight for each component can be computed by means of normalization, i.e., 100/185 is equal to 54.05% for the weight for the acrylic polymer emulsion resin, 8.11% for cement, 16.22% for silica powder, 0.54% for sodium sulfate and EPS, 4.32% for anhydrite, and 16.22% for fly ash. A mixing ratio of the resilient materials for each component is summarized in Table 2. The properties of the ingredient for the place-type resilient material are summarized in Table 3, Table 4, Table 5, Table 6, Table 7 and Table 8, respectively.

**Figure 1:**
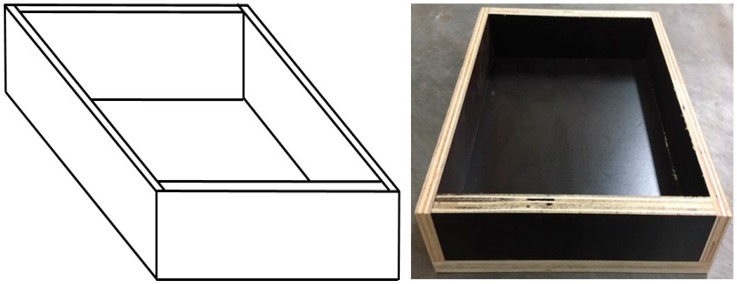
A mold for specimen of a place-type resilient material.

The specimens, having a square-shaped mold with dimensions 200 mm × 200 mm × 40 mm, as shown in Figure 1, were fabricated in order to assess the physical properties of the place-type resilient materials.

**Figure 2:**
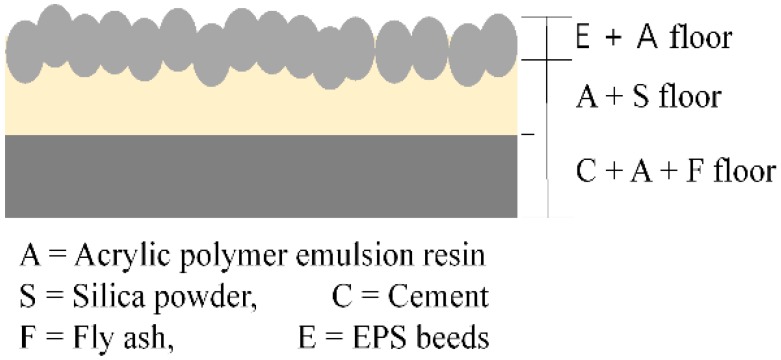
Schematic diagram of the place-type resilient material.

The mixture was created to switch from the conventional adhesion type to a new place-type. In order to satisfy the basic requirements for typical resilient materials, the proposed resilient materials, as shown in Figure 2, were fabricated based on the differences in the specific gravity to ensure that: (1) the cement and fly ash settle to the bottom and gain stiffness; (2) the resin and silica powder harden with elasticity in the intermediate layer; and (3) the EPS beads harden in a floating manner in the upper part.

**Figure 3:**
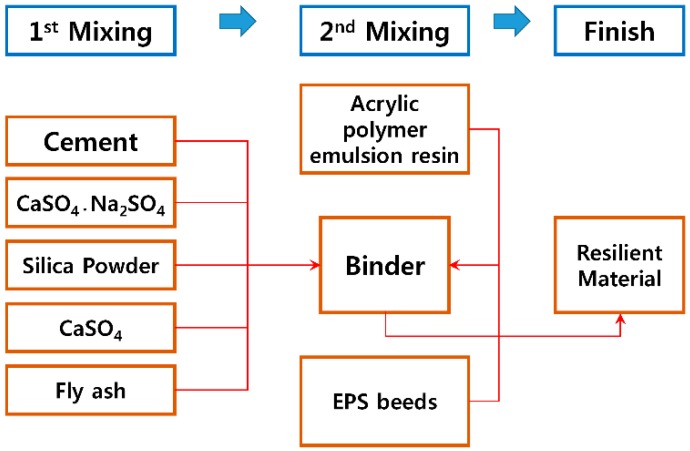
Experiment process of the place-type resilient material.

Figure 3 shows the experimental method, where mineral powder was mixed and stirred for 30 s, and the acrylic resin and mineral materials were mixed and stirred for 90 s. The materials in a slurry state and the EPS beads were mixed and stirred for an additional 30 s before being placed in the mold. The mixture was then dried and cured for 7 days, after which the density, dynamic stiffness, remanent strain and compressive strength were measured. It should be noted that the samples were stirred manually and dried at room temperature during the manufacturing process.

### 2.2. Measuring Methods

Measuring methods for the proposed place-type resilient materials are selected and carried out in accordance with ISO (International Organization for Standardization) and KS (Korean industrial Standards), respectively. For density testing, measurements were carried out using the mass and volume of each specimen in accordance with KS M 0602 [27], which is similar to ISO 2781 [28] or ISO 1183 [29]. For compressive strength testing, maximum weight was obtained with pressurization at the weight speed of 800 (±50) N/s using 3 samples with a weight board for weight in 40 mm × 40 mm × 40 mm in accordance with KS L 5105 [30], which is similar to ISO 679 [31].

**Figure 4:**
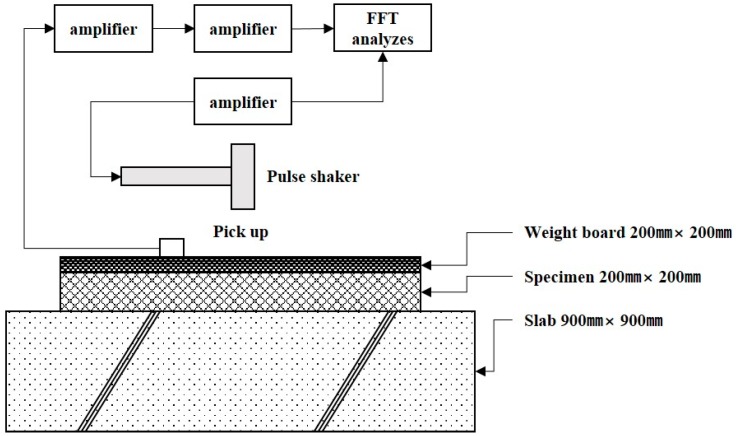
Test method for dynamic stiffness by pulse shaker.

For dynamic stiffness experiments, the center of a weight board was hit and shaken in a single shot with a pulse shaker under the pulse shake method as shown in Figure 4 in accordance with KS F 2868 [32], which is similar with ISO 9052-1 [33], and response wave forms of vibration velocity were measured for 1 point near the shake point, where an arithmetical mean was taken from 5 measurements in total.

**Figure 5:**
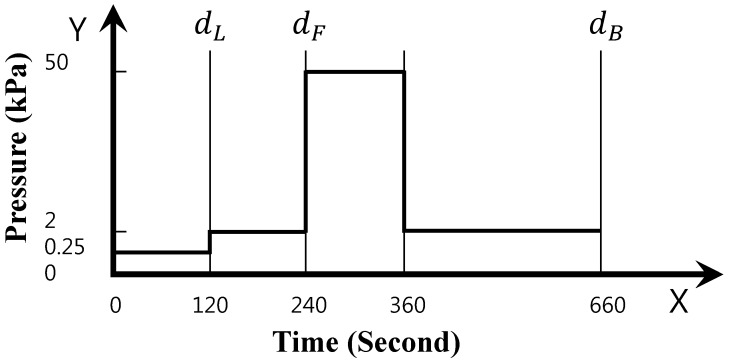
Measuring thickness according to time and weight for remanent strain.

For remanent strain, a weight was continuously added to the measured specimen for the defined time, as shown in Figure 5, using the method in accordance with KS F 2873 [34], which is similar to ISO 29770 [35]. The thickness of the specimen was measured after a certain time, and expressed in an arithmetical mean with the thickness before the weight was added.

### 2.3. Properties of the Place-Type Resilient Materials

**Figure 6:**
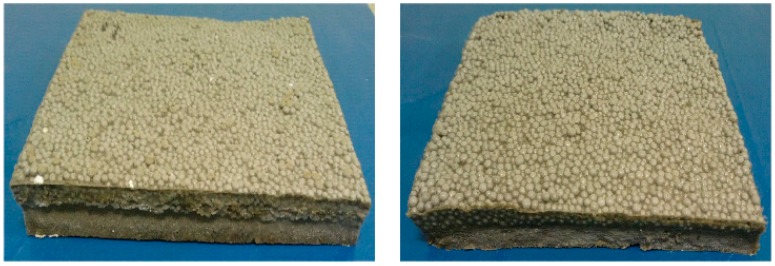
The proposed place-type resilient material.

Figure 6 shows the proposed place-type resilient materials. Overall, nine specimens are made using various combinations of the above-mentioned materials and experimental tests to evaluate the properties were conducted. The results are summarized in Table 9. Detailed information related to the properties of the proposed resilient materials follows. As shown in Figure 6, the surface of the specimens is uneven. Therefore, measurements have been carried out after making flat surface using gypsum-capping.

#### 2.3.1. Density

**Figure 7:**
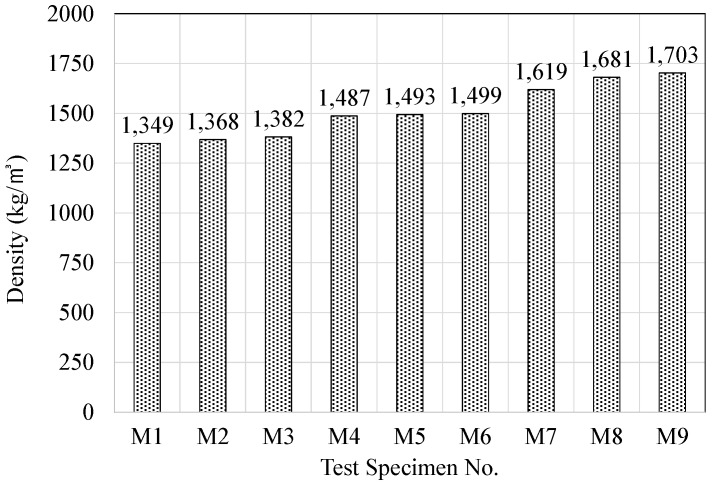
Variations of density according to mixing conditions.

The density of the resilient materials fabricated with acrylic resin and mineral binder increased substantially in the case of using cement as a mineral binder, as shown in Figure 7. An increase in the amount of silica powder resulted in a higher rate of increase in density. This is deemed to be due to the fact that cement has the highest specific gravity among the mineral materials that were used, and an increased amount of cement boosts the density of the internal matrix of the resilient material. The increase in density caused by an increased amount of silica powder is also thought to be due to the same reason.

#### 2.3.2. Compressive Strength

**Figure 8:**
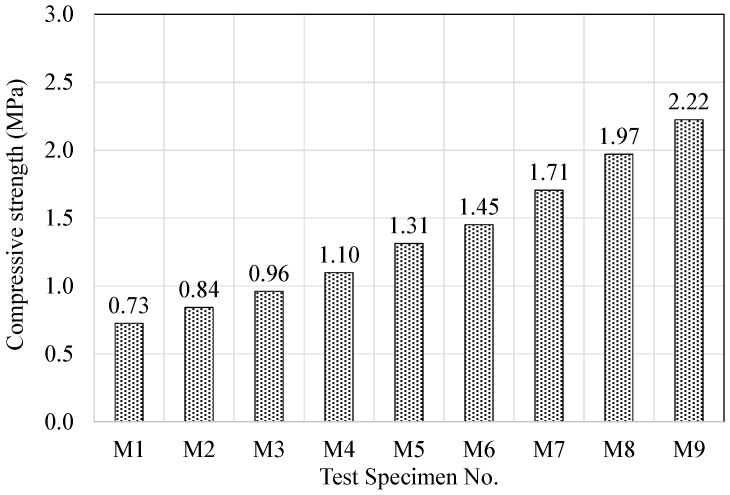
Variations of compressive strength according to mixing conditions.

The compressive strengths of the resilient materials fabricated using acrylic resin and mineral binders are shown in Figure 8. Specimens M1, M2, and M3, which did not contain any cement, showed the lowest compressive strength at 0.73, 0.84, and 0.96 MPa, respectively, whereas specimens M7, M8, and M9, which had the highest cement content, showed the highest strength at 1.71, 1.97, and 2.22 MPa, respectively. In addition, specimens M4, M5, and M6, which had 15% cement content, showed strengths of 1.10, 1.31, and 1.45 MPa, respectively. It is commented that the strength of the specimens having 15% cement content is lower than that of the specimens having 30% cement content.

It was found that an increase in the quantity of cement per unit and the silica powder replacement ratio led to a boost in strength. This is deemed to be caused by an increase in the filler inside the matrix during the hardening of acrylic resin, which was used as a binder and granted elasticity to the resilient materials.

The acrylic resin used in the fabrication of the resilient materials was waterborne acrylic polymer emulsion resin with 35% water content. Thus, in the case of adding cement, the acrylic resin hardens due to evaporation and the water is consumed in the cement hydration process. The acrylic resin also adsorbs the cement hydrates and fills the voids in the hydrates, thereby enhancing the strength and adhesive property of the cement paste and grants elasticity to the cement matrix. This can be observed in the results of the dynamic stiffness and remanent strain. In addition, specimens M7, M8, and M9 contain high percentages of materials such as cement, fly ash and sodium sulfate that directly contribute to strength enhancement, and it is deemed that when such materials are mixed with acrylic resin, a hydration reaction occurs between the resin and binder, thereby enhancing strength. In addition, the results were also consistent with the general reports of a proportional relationship between density and compressive strength [36].

#### 2.3.3. Dynamic Stiffness

**Figure 9:**
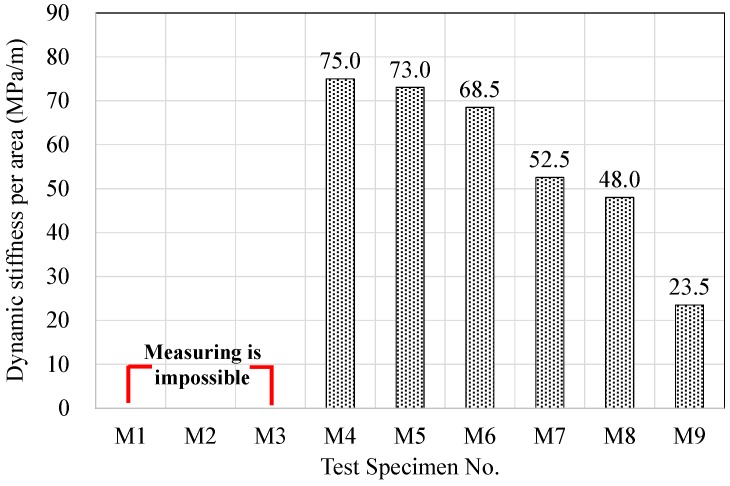
Variations of dynamic stiffness according to mixing conditions.

The dynamic stiffness per area values for the resilient materials made using acrylic resin and mineral binders are shown in Figure 9. In the case of specimens M1, M2, and M3, which did not contain any cement, it was impossible to measure the dynamic stiffness. This is because the sinusoidal excitation techniques, based on ISO 9052-1, were used to measure the dynamic stiffness of specimens. During the laboratory measurements, the oscillation period required to evaluate the dynamic stiffness of the specimens was not measured. Therefore, as shown in Figure 9, the dynamic stiffness of specimens M1, M2, and M3 could not be measured.

It should be noted that the samples having the higher amount of cement present the lower values of dynamic stiffness due to the increasing the density and hardening the matrix process of the samples. For the rest of the specimens, it showed that the less silica powder the specimen has, the higher its dynamic stiffness becomes for each cement ratio case. Therefore, dynamic stiffness was found to be positively correlated with the mineral binder content. Generally, dynamic stiffness is known to be significantly influenced by material density, and the results of this experiment showed similar tendencies [37].

#### 2.3.4. Remanent Strain

**Figure 10:**
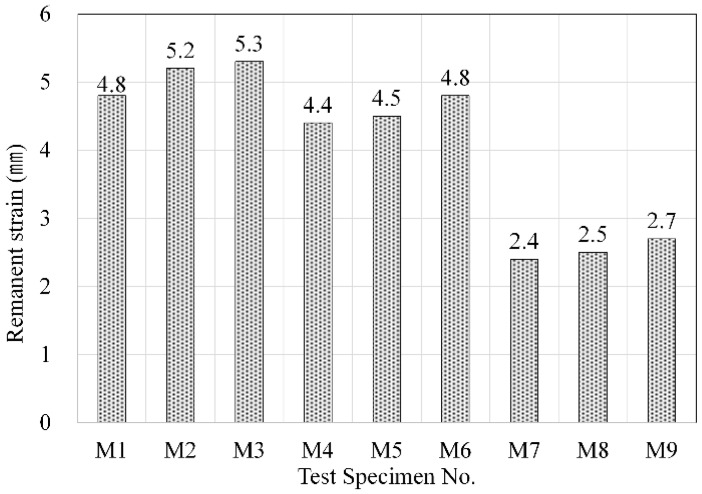
Variations of remanent strain according to mixing conditions.

The remanent strains of the resilient materials made using acrylic resin and mineral binders is shown in Figure 10. In the case of specimens M1, M2, and M3, which did not contain any cement, the remanent strain was found to be 4.8, 5.2, and 5.3 mm, respectively, which were the highest among all specimens. In these specimens, the acrylic resin accounted for most of the matrix hardening process, and they had the highest elasticity after the hardening process. Accordingly, this is the reason these specimens exhibited the largest degrees of deformation when load was applied. It should be noted that the samples having lower amount of cement present the higher remanent strain due to revealing the lower dynamic stiffness and the lower compressive strength. On the other hand, remanent strain decreased with an increase in the cement content. Because the remanent strain test demonstrates the displacement resulting from an upper load applied to a specimen, the remanent strain was shown to decrease with higher density and strength, and this was also observed in this experiment.

## 3. Evaluation of Floor Impact Noise Reduction

### 3.1. Experimental Tests on the Floor

To evaluate the floor impact noise reduction, an experimental test was carried out. To do this, four specimens have been selected based on the test results of the properties. Specimens M1, M2, and M3, which did not contain any cement, have been excluded due to their low compressive strength. Two mixing ratio for each cement and silica powder, i.e., 15% and 30% for cement, and 25% and 35% for silica powder, were considered. It should be noted that experimental test for floor impact requires a long construction process time, including manufacturing the resilient materials, constructing floor system, installing resilient materials in the floor system, etc. Therefore, the 30% mixing ratio of silica powder, which is the middle values of the three mixing ratios, because of the linear relationship among the three mixing ratios for silica powder based on the testing results of the properties, was excluded. Finally, four specimens out of six have been chosen due to the limited budget and manufacturing and testing time. The experimental factors were set as the five types of resilient material and the two types of floor impact source. Table 10 shows the mixing ratios for the resilient materials used in this experiment. Floor impact noise was measured in a test residential structure.

**Figure 11:**
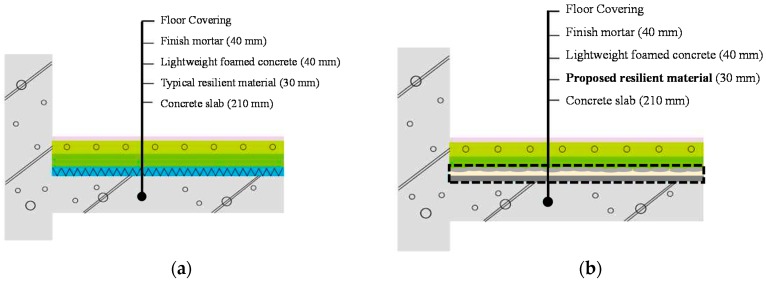
Schematic overview of floor system with resilient materials installation: (**a**) typical floor system; and (**b**) proposed floor system.

To simulate the noise caused mainly by walking, running, and jumping on the above floor in multi-unit dwelling residential structures, standard heavy-weight floor impact sources such as the bang machine and the impact ball are adopted in this study [38,39,40,41,42]. The structure adopted in this study employed a box-frame type structural system to mimic a typical multi-unit dwelling for practical experimental purposes. Figure 11 depicts the details of a typical floor type in multi-unit residential structures in South Korea. The floor system consists of five layers, floor covering layer, finish mortar layer, lightweight foamed concrete layer, resilient material, and concrete slab layer.

To perform the test, the floor systems are to be measured for floor impact noise after placing the resilient material, foamed concrete and finish mortar on the concrete slab, as shown in Figure 11a. However, the placing-type resilient material developed in this study was placed on a concrete slab to a thickness of 40 mm, as shown in Figure 11b, and cured at room temperature for seven days before pouring cast-in-place foamed concrete to a thickness of 40 mm.

**Figure 12:**
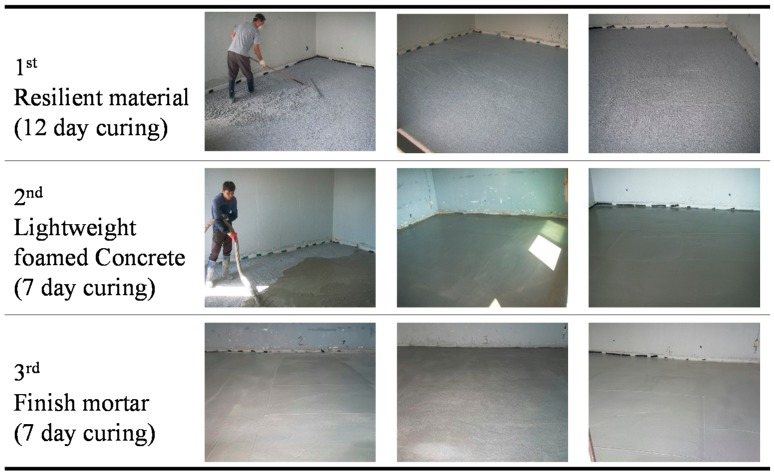
Construction process for floor system using resilient materials.

Figure 12 shows the field construction work involved. Firstly, the foamed concrete was cured at room temperature for 12 days to ensure sufficient hardening, and the finish mortar was laid to a thickness of 40 mm afterwards. Then, the floor structure, i.e., resilient materials, lightweight foamed concrete, and finishing mortar, was then placed in an acoustics test building, which had been built to allow tests to be conducted on the sound impact noise insulation performance for multi-family residential housing. It should be noted that, typically, the acoustics test building has only concrete slab, so there is no curing period for concrete slab. As mentioned before, 12 day is needed to cure the resilient materials, seven days for lightweight foamed concrete, and seven days for finishing mortar. Therefore, 26 days for curing periods is needed to construct a floor system.

**Figure 13:**
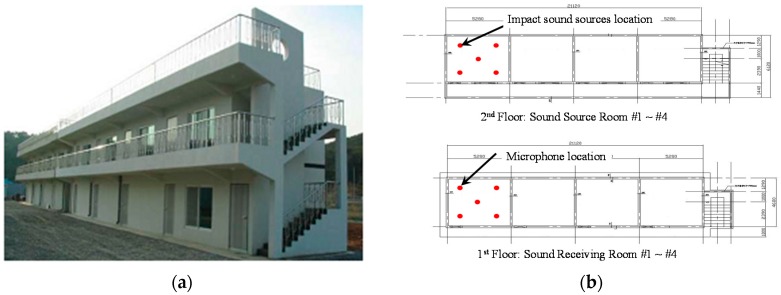
Overview and floor plan of the test building: (**a**) ISO view of the test building; and (**b**) floor plan of the test building.

As shown in Figure 13, four rooms of the same size (64.26 m^3^, or 4.5 m × 5.1 m × 2.8 m) were used. The place-type resilient material fabricated in this study was installed during construction. The conventional EPS resilient material manufactured by SIP Co., Seoul, Korea, that is denoted as default material for a comparison, were applied.

### 3.2. Natural Frequencies of the Floor System

The theoretical natural frequencies of the floor system with the proposed resilient materials, depicted in Figure 11, have been computed to investigate the relationship between the natural frequency and the resilient materials. From Figure 11, the floor system can be idealized as two-plate system. One plate, denoted as the floating plate, consists of finishing mortar and lightweight foamed concrete. It should be noted that the floor covering is typically installed but is not considered during measurements. The other plate, denoted as the base plate, is the concrete slab. It should be noted that there is floor covering in Figure 11. The mass of common floor covering material is tiny. Therefore, it has not been considered during the frequency calculation process. 

The floor system with the resilient materials can be assumed to be the two-plate system. The natural frequency of the two-plate system at low-frequencies range, i.e., typically less than 1000 Hz, can be defined as [21,22]:

(1) $$f_{n}=\frac{1}{2\pi }\sqrt{s^{\prime }\left(\frac{1}{m_{1}^{\prime }}+\frac{1}{m_{2}^{\prime }}\right)}$$

where *f_n_* is the natural frequency, *s’* is the dynamic stiffness of the interlayer, i.e., resilient material in this study, and *m*_1_*’* and *m*_2_*’* are the areal densities of the floating and base plates, respectively.

Material properties of the floor system with the proposed resilient materials [3,22] are tabulated in Table 11. The natural frequency has been computed using Equation (1) and tabulated in Table 12. 

### 3.3. Floor Impact Sound Experiment

The floor impact noise experiment was conducted in accordance with KS F 2810 [41] that is very similar with ISO 16283 [40]. For storage of the measurement data, a laptop and sensor signal acquisition device (Front-End SIEMENS SCADAS Mobile, SIEMENS, Plano, TX, USA), Microphone (4188, Brüel & Kjær, Nærum, Denmark), and Preamplifier (2671, Brüel & Kjær, Nærum, Denmark) were used. It prescribes that there be a total of five impact sources including four spots along the edges that are 0.75 m away from the wall and one central spot in the sound source room, and that a microphone be installed at each of the five points including the four spots on the edges and one central spot in the sound receiving room (Figure 13). With the impact ball and bang machine used as the impact sound sources, the sound pressure level (SPL) of floor impact noise is to be measured, accordingly. It should be noted that the tapping machine as light weight impact sound source and the rubber ball as heavy weight impact sound source are allowed in ISO 16283. However, the bang machine is only allowed as heavy weight impact source KS F 2810, the rubber ball is excluded recently [43]. However, two impact sources, i.e., the bang machine and the rubber ball, as heavy weight impact sound source are all taken into consideration. Thus, these standards were applied to this experiment for the purpose of measuring and analyzing the floor impact noise.

**Figure 14:**
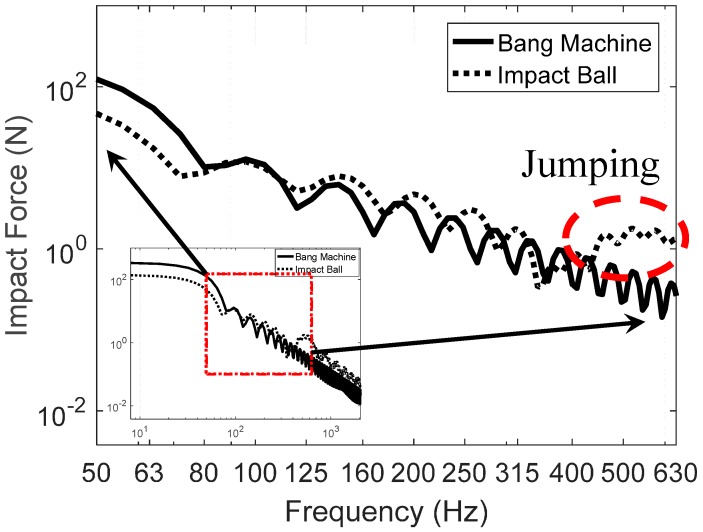
Two floor impact sources.

Figure 14 shows impact ball and bang machine that are numerically simulated y using the analytical impact force model proposed by Park and Kim [23]. From Figure 14, there is a jumping around 400 Hz in impact ball force. In addition, the trends of both impact force sources show that the magnitude of each hill is decreased as frequency is increased.

Firstly, impact ball was used as a floor impact source. Table 13 and Table 14 show that the SPL of the proposed resilient material expressed in 1/3 Octave bands and 1/1 Octave bands, respectively. From the results, the acoustic performance of some specimens is better than that of the specimen base. Especially, all specimens have reduced the floor impact noise level comparing with the specimen base in the range from 125 Hz to 250 Hz. Specimen no. R4, i.e., the mix ratio of 30% cement and 35% silica powder, has the best performance in floor impact noise reduction and it reduced 3 dB in single number quantity (SNQ) from 50 dB to 47 dB. It should be noted that the SNQ in accordance with ISO 717-2 [44] is intended for rating impact sound insulation of floor assemblies using the standard tapping machine, which is lightweight floor impact source. On the contrary, the SNQ of heavyweight floor impact source, i.e., bang machine and impact ball, is needed to evaluate impact sound insulation of floor assemblies in this study. Therefore, the SNQ is computed in accordance with KS F 2863-2 [45] that defines how to compute the SNQ with respect to the heavyweight floor impact sources. The only difference between ISO 717-2 and KS F 2863-2 is the frequency range to be considering for computation. To evaluate single number quantity in accordance with ISO 717-2, values obtained in accordance with ISO 10140-3 are needed. The values are then compared with reference values at the frequencies of measurements within the range 100 Hz to 3150 Hz for measurements in 1/3 Octave bands or 125 Hz to 2000 Hz for measurement in 1/1 Octave bands. The reference curves in increments of 1 dB are then shifted towards the measured curve until the sum of unfavorable deviations is as large as possible but not more than 32 dB for 1/3 Octave bands and 10 dB for 1/1 Octave bands. The SNQ is termed as normalized impact sound pressure level, denoted as $L_{n}$. If the frequencies of measurements within the range 100 Hz to 500 Hz for measurements in 1/3 Octave bands or 125 Hz to 500 Hz for measurement in 1/1 Octave bands is considered then the sum of unfavorable deviations is not more than 10 dB. In this case, the symbol of SNQ is $L_{n}^{\prime }$. The procedure for evaluating the SNQ in KS F 2863-2 is identical to ISO 717-2. The only difference between ISO 717-2 and KS F 2863-2 is that KS F 2863-2 considers the frequencies of measurements within the range 63 Hz to 500 Hz for measurements in 1/1 Octave bands, i.e., 63 Hz, 125 Hz, 250 Hz, and 500 Hz, and the sum of unfavorable deviations is as large as possible but not more than 8 dB for 1/1 Octave bands.

Overall performance of the proposed resilient materials according to the mixing ratio conditions is an augmentation below 100 Hz in all specimens. On the contrary, there is a reduction in the range from 125 Hz to 315 Hz. After 400 Hz, SPL has been increased again compared to the specimen base. As can be seen in Figure 14, the magnitude of the impact ball has increased after 400 Hz, which can explain this augmentation. Additionally, specimens no. R1 and no. R2 have the same amount of cement replacement ratio but the difference ratio for silica powder. Therefore, the mixing ratios of the cement for specimen no. R1 and no. R2 are different (see Table 2). Due to the different amounts of cement and silica powder, their behaviors are different, leading to a 6.5 MN/m^3^ difference in dynamic stiffness and 7 Hz in natural frequency. Finally, this results in the 3 dB difference in SNQ. One can argue that there are many differences of material components between the two specimens, but the cement and silica powder are the main components in this study. These differences were found again for the comparison between specimens no. R3 and no. R4. Based on the results, it was deemed that an increase in the cement content reduced the level of impact noise a certain amount, as did the silica powder content.

**Figure 15:**
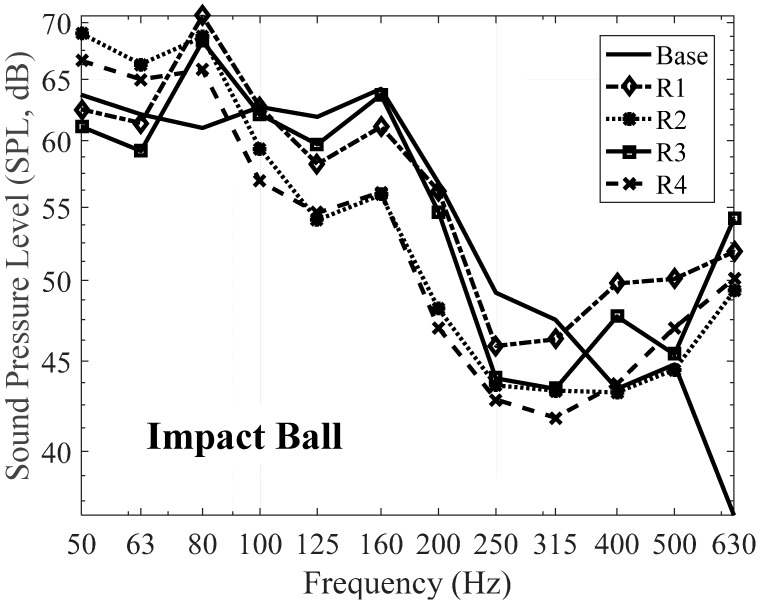
Floor impact sound level with the proposed materials in 1/3 Octave bands—Impact ball.

**Figure 16:**
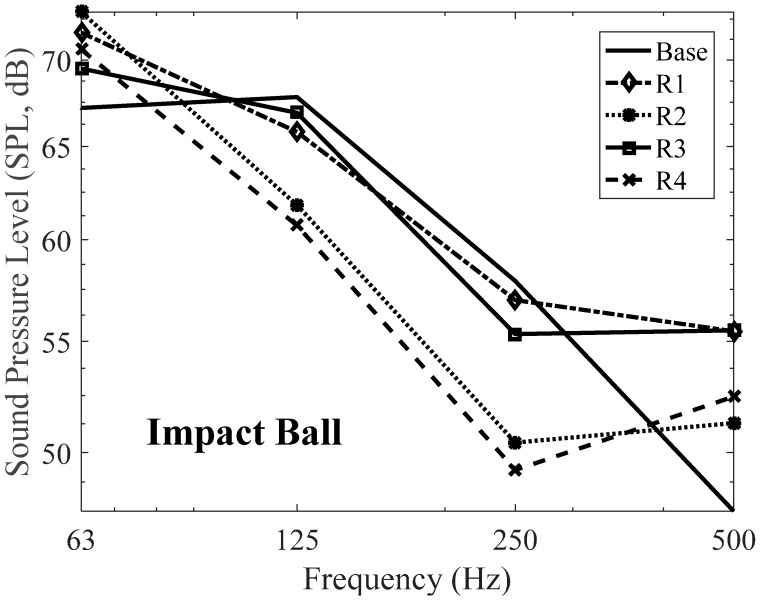
Floor impact sound level with the proposed materials in 1/1 Octave bands—Impact ball.

Figure 15 and Figure 16 show the heavyweight impact noise level by impact ball as an impact source expressed in 1/3 Octave bands and 1/1 Octave bands, respectively, when the proposed resilient materials were applied to a floor structure. Compared to the specimen base, specimen no. R4 exhibited a characteristic of reducing floor impact noise level. Generally, it has been reported that a low dynamic stiffness of a resilient material results in high floor impact noise reduction, and this was also the case in this experiment, with high noise reduction observed with low dynamic stiffness, high density and strength, and low remanent strain. In the case of the specimen with low density and strength and high remanent strain, for instance the specimen base, the impact noise level rose due to resonance around 63 Hz, despite having low dynamic stiffness [46,47]. This is deemed to have been caused by an amplification effect in the low-frequency bands such as 63 Hz, as there are resonance effects in the area of adhesion among the resilient materials, the concrete slab, foamed concrete, and finish mortar. These behaviors and characteristics are explained by the performance of the specimen base, i.e., dynamic stiffness of the specimen base is relatively low comparing with the four specimens, but its performance is not always better than others.

On the other hand, it has been reported that there is strong relationship between the natural frequency and the acoustic behavior. For instance, specimen no. R1 has 156.27 Hz as the natural frequency and there are two augmentations around 80 Hz and 160 Hz. It can be explained that these augmentations could be related to either the resonance of the impact source or the resonance of the natural frequency of the material itself. This situation can be applied to other specimens, i.e., around 160 Hz for specimen no. R2 and no. R3, and around 80 Hz for specimen no. R4.

It must be commented that there is an augmentation after 400 Hz for all four specimens. There is a minor increment in the specimen base after 400 Hz but its trend shows the decrements of the SPL as increasing the frequency. On the contrary, all four specimens have the same behavior of the specimen base but the magnitude of the SPL is relatively high. From Figure 14, the magnitude of the impact ball has increased after 400 Hz, which can explain this augmentation. Additionally, this could be because the components of the proposed resilient materials and their mixing ratios are different with the specimen base, so similar behaviors with higher magnitude after 400 Hz were investigated.

Secondly, bang machine was used as a floor impact source and measuring was performed. Table 15 and Table 16 show the SPL of the proposed resilient materials expressed in 1/3 Octave bands and 1/1 Octave bands, respectively. Only the acoustic performance of specimen no. R4 is better than that of the specimen base. Specimen no. R4 has the reduction level of 1 dB in the SNQ from 51 dB to 50 dB. 

**Figure 17:**
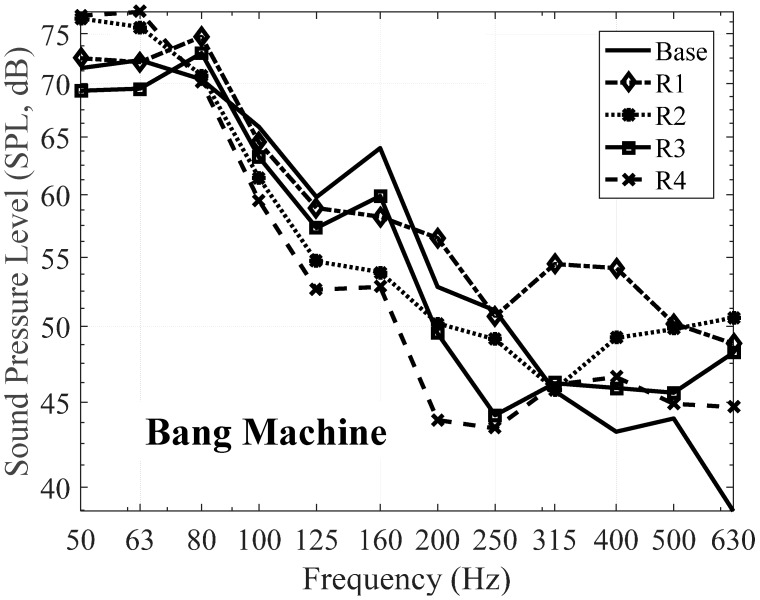
Floor impact sound level with the proposed materials in 1/3 Octave bands—Bang machine.

**Figure 18:**
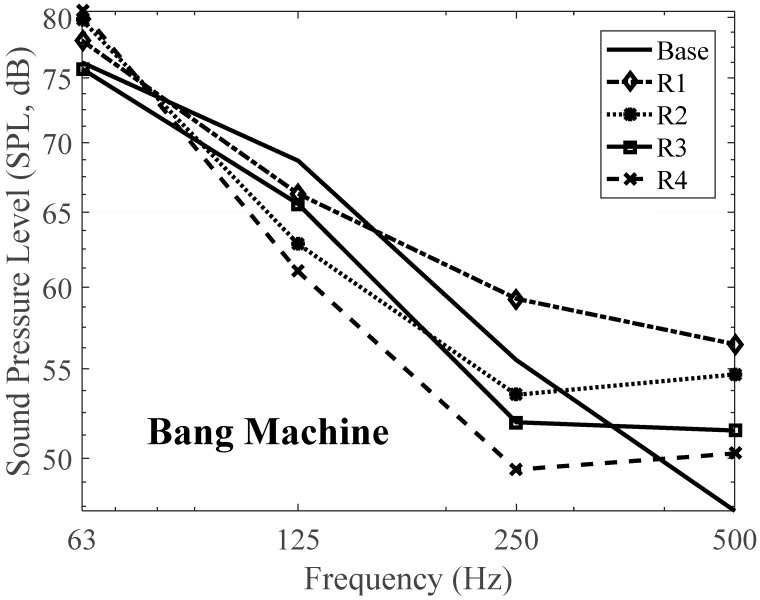
Floor impact sound level with the proposed materials in 1/1 Octave bands—Bang machine.

Figure 17 and Figure 18 shows the results by bang machine as an impact source expressed in 1/3 Octave bands and 1/1 Octave bands, respectively. It was found that the reduction of the impact noise level was lower than when that of the impact ball was used. Additionally, the SNQ, which is the unique number used to evaluate the acoustic performance of the material, of the impact ball was lower than that of the bang machine. For this reason, the impact ball was excluded from floor impact noise measurements recently [23,48]. The results of using the bang machine were similar to using the impact ball. In the case of the specimen with relatively low density and strength and high dynamic strength, it showed amplified heavyweight impact noise in the low-frequency areas such as 125 Hz and 250 Hz. However, the amplification effect and the impact noise levels became reduced at higher frequencies, which is very similar behavior when the impact ball was used. In the case of the specimens with increased amounts of cement and silica powder, the impact noise levels were reduced in the low-frequency bands.

In the case of specimen no. R2 and no. R4, the impact noise levels were lower in all of the frequency bands ranging from 80 Hz to 315 Hz. As mentioned earlier, there is an augmentation after 400 Hz for two specimens, i.e., specimen no. R2 and no. R3. The other two specimens, no. R1 and no. R4, decreased as the frequency increased. In addition, the increments at 160 Hz in Figure 17 were investigated and it could be explained due to the resonance of the material itself as mentioned before.

## 4. Summary and Conclusions

New place-type resilient materials were fabricated using acrylic resin and mineral binders for the purpose of mitigating the floor impact noise issue in multi-unit residential buildings in South Korea. Experiments were conducted for material properties and acoustic performances of the proposed place-type resilient materials.Nine specimens for material properties testing and five specimens, four specimens from the proposed resilient materials and one from conventional EPS resilient material as the specimen base case, for acoustic performance testing are considered during the experiments. Four specimens from nine specimens were chosen for the floor impact experiments based on the testing results of the material properties. Relationship between the mixing ratios of cement and silica powder, and material properties such as density, compressive strength, dynamic stiffness, and remanent strain were investigated.

Floor impact noise was measured in a test residential building. Two standard heavyweight floor impact sources, the bang machine and the impact ball, were adopted to simulate the noise. Considering the experimental results, specimen no. R4 is the best product to obtain overall performance for both impact ball and bang machine. It has reduced the SNQ level by 3 dB for the impact ball and 1 dB for the bang machine.

The results of assessing their physical properties and floor impact noise reduction effects are as follows:

(1) In the case of resilient materials fabricated with acrylic resin and mineral binders, an increase in the cement and silica powder replacement ratios caused an increase in density due to the high specific gravity of the raw materials, and an increase in density in turn reduced the dynamic stiffness.
(2) Resilient materials with high cement and silica powder replacement ratios developed high strength due to the hydration characteristics and promotion effect of the raw materials.
(3) Remanent strain was found to be inversely proportional to density and strength. For instance, specimens with high density and strength had low remanent strain.
(4) Heavyweight impact noise measurements showed that materials with high density and strength and low remanent strain exhibited high noise reduction rates, and that low dynamic stiffness was also advantageous in reducing the impact noise.
(5) The acoustic performance of the materials with low density, low strength, and high remanent strain does not always result in reducing impact nose level due to the resonance around 63 Hz and its effects in the area of adhesion among the resilient material, the concrete slab, foamed concrete, and finish mortar.

In conclusion, the new place-type resilient material proposed in this study demonstrated the possibility of reducing the floor impact noise. The developed resilient materials are able to satisfy the requirements given by the revised standards [4]. The acoustic performance of the floor system with the developed resilient materials is rated as a fourth level, which is the range between 47 dB and 50 dB, under the heavyweight impact source. From the results, it can be helpful to reduce the floor impact noise by means of using new resilient materials. Additionally, there needs to be further research to determine the ideal type of acrylic resin, mixing ratio, shape and thickness of the place-type resilient material, as well as the lightweight noise reduction effects.

## Figures and Tables

**Table 1:** Replacement ratio for each component.

Case No.	Cement (%)	Silica Powder (%)	Sodium Sulfate (%)	EPS (%)	Anhydrite (%)	Fly Ash (%)
M1	0	25	1	1	8	30
M2		30				
M3		35				
M4	15	25				
M5		30				
M6		35				
M7	30	25				
M8		30				
M9		35				

**Table 2:** Mixing ratio of resilient materials for experiments.

Case No.	Acrylic Polymer Emulsion Resin (%)	Cement (%)	Silica Powder (%)	Sodium Sulfate (%)	EPS (%)	Anhydrite (%)	Fly Ash (%)
M1	60.60	0.00	15.15	0.61	0.61	4.85	18.18
M2	58.82	0.00	17.65	0.59	0.59	4.70	17.65
M3	57.14	0.00	20.00	0.57	0.57	4.57	17.15
M4	55.55	8.33	13.89	0.56	0.56	4.44	16.67
M5	54.05	8.11	16.22	0.54	0.54	4.32	16.22
M6	52.63	7.89	18.42	0.53	0.53	4.21	15.79
M7	51.28	15.39	12.82	0.51	0.51	4.10	15.39
M8	50.00	15.00	15.00	0.50	0.50	4.00	15.00
M9	48.78	14.63	17.08	0.49	0.49	3.90	14.63

**Table 3:** Properties of acrylic polymer emulsion resin.

Density (kg/m^3^)	Dissoluble Solid (%)	Viscosity (cps)	Tg (°C)	Water Content	State
1040 ± 100	50.0 ± 1.0	10~800	−15	35%	liquid

**Table 4:** Properties of cement.

Density (kg/m^3^)	Blaine Fineness (m^2^/kg)	44 μm on Residue (%)	Setting Time (min)		Compressive Strength (MPa)		
			Initial	Final	3 Days	7 Days	28 Days
3140	320	12.5	240	370	22.5	30.0	39.5

**Table 5:** Properties of fly ash.

SiO_2_ (%)	Water (%)	LOI (%)	Density (kg/m^3^)	Blaine Fineness (m^2^/kg)	Flow Value Ratio (%)	Activity Index (%)
48.8	0.1	3.5	2140	336	101	81

**Table 6:** Chemical compositions of anhydrite (unit: %).

SiO_2_	Al_2_O_3_	Fe_2_O_3_	CaO	MgO	Na_2_O	K_2_O	SO_3_	Total
2.5	0.08	0.10	39.1	0.67	–	–	57.2	99.65

**Table 7:** Properties of silica powder.

Purity (%)	Size (µm)	Blaine Fineness (m^2^/kg)	Loss on Ignition (wt %)	pH	Hardness (Mohs)	Density (kg/m^3^)
99.8	14.1	2000	3	7	7.0	1350

**Table 8:** Properties of EPS beads.

Density (kg/m^3^)	Absorption Ratio (%)	Size (mm)	Color
350	0	2.9	White

**Table 9:** Test results of the properties of the place-type resilient materials.

Case No.	Density (kg/m^3^)	Compressive Strength (MPa)	Dynamic Stiffness (MN/m^3^)	Remanent Strain (mm)
M1	1349.38	0.73	–	4.8
M2	1368.13	0.84	–	5.2
M3	1381.88	0.96	–	5.3
M4	1486.88	1.10	75.0	4.4
M5	1493.13	1.31	73.0	4.5
M6	1498.75	1.45	68.5	4.8
M7	1619.38	1.71	52.5	2.4
M8	1680.63	1.97	48.0	2.5
M9	1702.50	2.22	23.5	2.7

**Table 10:** Mix ratio of resilient materials for floor impact noise testing.

Case No.	Cement (%)	Silica Powder (%)	Resin	Test Specimen No.
Base	Conventional EPS resilient material			–
R1	15	25	Acrylic polymer emulsion resin	M4
R2		35		M6
R3	30	25		M7
R4		35		M9

**Table 11:** Material properties of the idealized two-plate system.

Name	Material	Thickness (mm)	Density (kg/m^3^)	$m_{i}^{’}$ (kg/m^2^)
Floating Plate	Finishing mortar	40	1800	92 (72 + 20)
	Lightweight foamed concrete		500	
Base Plate	Concrete Slab	210	2400	504

**Table 12:** Natural frequency of resilient materials.

Material	Dynamic Stiffness ($s^{’}$, MN/m^3^)	Natural Frequency (Hz)
Base	10.0	57.06
R1	75.0	156.27
R2	68.5	149.34
R3	52.5	130.74
R4	23.5	87.47

**Table 13:** Impact ball’s test results in 1/3 Octave bands.

Frequency (Hz)	Case No. (dB)				
	Base	R1	R2	R3	R4
50	63.7	62.5	69.0	61.1	66.6
63	62.1	61.4	66.3	59.2	65.0
80	61.0	70.6	68.7	68.3	65.8
100	62.7	62.7	59.4	62.1	56.9
125	61.9	58.2	54.1	59.7	54.6
160	64.2	61.1	55.9	63.7	56.1
200	56.8	56.2	48.2	54.7	47.0
250	49.2	45.9	43.6	44.0	42.8
315	47.5	46.3	43.3	43.4	41.8
400	43.4	49.8	43.2	47.7	43.7
500	44.8	50.1	44.5	45.4	47.0
630	36.8	51.9	49.3	54.2	50.1

**Table 14:** Impact ball’s test results in 1/1 Octave bands.

Frequency (Hz)	Case No. (Difference, dB)				
	Base	R1	R2	R3	R4
63	67.2	71.7(+4.5)	72.9(+5.7)	69.5(+2.3)	70.6(+3.4)
125	67.8	65.8(−2.0)	61.8(−6.0)	66.9(−0.9)	60.7(−7.1)
250	57.9	57.0(−0.9)	50.4(−7.5)	55.3(−2.6)	49.3(−8.6)
500	47.6	55.5(+7.9)	51.3(+3.7)	55.5(+7.9)	52.5(+4.9)
SNQ ($L_{i,Fmax,AW})$	50	51	48	51	47

**Table 15:** Bang machine’s test results in 1/3 Octave bands.

Frequency (Hz)	Case No. (dB)				
	Base	R1	R2	R3	R4
50	71.5	72.5	76.6	69.3	76.9
63	72.3	72.1	75.7	69.5	77.3
80	70.4	74.7	70.8	73.0	70.1
100	65.9	64.5	61.4	63.2	59.5
125	59.8	58.9	54.7	57.3	52.6
160	64.0	58.2	53.9	59.9	52.8
200	52.8	56.5	50.2	49.5	43.9
250	51.1	50.7	49.1	44.2	43.4
315	45.7	54.5	45.8	46.2	46.1
400	43.2	54.2	49.2	45.9	46.6
500	44.0	50.2	49.8	45.6	44.9
630	38.7	48.8	50.6	48.2	44.7

**Table 16:** Bang machine’s test results in 1/1 Octave bands.

Frequency (Hz)	Case No. (Difference, dB)				
	Base	R1	R2	R3	R4
63	76.2	78.0(+1.8)	79.8(+3.6)	75.7(−0.5)	80.5(+4.3)
125	68.7	66.3(−2.4)	62.8(−5.9)	65.6(−3.1)	61.0(−7.7)
250	55.5	59.3(+3.8)	53.5(−2.0)	52.0(−3.5)	49.4(−6.1)
500	47.3	56.5(+9.2)	54.7(+7.4)	51.5(+4.2)	50.3(+3.0)
SNQ ($L_{i,Fmax,AW})$	51	53	52	50	50

## Abbreviations

The following abbreviations are used in this manuscript:

EPS	Expanded Polystyrene
EVA	Ethylene Vinyl Acetate
LOI	Lost on Ignition
ISO	International Organization for Standardization
KS	Korean industrial Standards
SPL	Sound Pressure Level
SNQ	Single Number Quantity
